# Therapeutic Potential of RTA 404 in Human Brain Malignant Glioma Cell Lines via Cell Cycle Arrest via p21/AKT Signaling

**DOI:** 10.1155/2021/5552226

**Published:** 2021-03-08

**Authors:** Tai-Hsin Tsai, Ann-Shung Lieu, Yi-Wen Wang, Sheau-Fang Yang, Yi-Chiang Hsu, Chih-Lung Lin

**Affiliations:** ^1^Division of Neurosurgery, Department of Surgery, Kaohsiung Medical University Hospital, Kaohsiung, Taiwan; ^2^Department of Surgery, School of Medicine, College of Medicine, Kaohsiung Medical University, Kaohsiung, Taiwan; ^3^Graduate Institute of Medicine, College of Medicine, Kaohsiung Medical University, Kaohsiung, Taiwan; ^4^Department of Nursing, Kaohsiung Medical University Hospital, Kaohsiung, Taiwan; ^5^Department of Pathology, Kaohsiung Medical University Hospital, Kaohsiung, Taiwan; ^6^Department of Pathology, School of Medicine, College of Medicine, Kaohsiung Medical University, Kaohsiung, Taiwan; ^7^School of Medicine, I-Shou University, Kaohsiung, Taiwan

## Abstract

**Background:**

Glioblastoma multiforme (GBM) is the most common malignant brain tumor in the world. Despite advances in surgical resection, radiotherapy, and chemotherapy, GBM continues to have a poor overall survival. CDDO (2-cyano-3,12-dioxoolean-1,9-dien-28-oic acid), a synthetic triterpenoid, is an Nrf2 activator used to inhibit proliferation and induce differentiation and apoptosis in various cancer cells. One new trifluoroethylamide derivative of CDDO, RTA 404, has been found to have increased ability to cross the blood-brain barrier. However, it is not clear what effect it may have on tumorigenesis in GBM.

**Methods:**

This in vitro study evaluated the effects of RTA 404 on GBM cells. To do this, we treated GBM840 and U87 MG cell lines with RTA 404 and assessed apoptosis, cell cycle, cell locomotion, and senescence. DNA content and induction of apoptosis were analyzed by flow cytometry and protein expression by Western blot analysis.

**Results:**

RTA 404 significantly inhibited the proliferation of tumor cells at concentrations higher than 100 nM (*p* < 0.05) and reduced their locomotion ability. In addition, treatment with RTA 404 led to an accumulation of RTA 404-treated *G*_2_/*M* phase cells and apoptosis. An analysis of the p21/AKT expression suggested that RTA 404 may not only help prevent brain cancer but it may also exert antitumor activities in established GBM cells.

**Conclusion:**

RTA404 can inhibit proliferation, cell locomotion, cell cycle progression, and induce apoptosis in GBM cells in vitro, possibly through its inhibition of N-cadherin and E-cadherin expression via its inhibition of the AKT pathway.

## 1. Introduction

One of the most invasive and malignant cancers in the modern world is Previorian cancer for which little has been developed in the way of chemotherapy [1, 2]. Glioma, the most common and malignant of these brain tumors, is classified by The World Health Organization (WHO) into four grades based on histologic features [3, 4]. WHO grade IV, also called glioblastoma multiforme (GBM), is angiogenic and can cause necrosis. Glioblastoma is an aggressive tumor of the central nervous system. Although much has been achieved in surgical treatment, radiotherapy, and chemotherapy for this disease, prognosis remains poor. Patients with glioblastoma have a median survival rate of about twelve months [5]. Thus, it is important to identify new biomarkers and therapeutic targets to help diagnose and treat this disease. Induction of apoptosis and arrest of the cell cycle are two of the best approaches to suppressing cancerous tumors [6]. This has been reported to have been achieved by phytochemicals synthesized from plants [7].

One component found in Chinese herbal medicine for hepatitis, oleanolic acid, has been modified chemically to create an oleanane triterpenoid,2-cyano-3-,12-dioxoolean-1,9-dien-28-oic acid (CDDO), a plant synthetized drug [8]. A master activator of antioxidant transcription factor nuclear factor erythroid 2-related factor 2 (Nrf2), CDDO, has been found to impede the synthesis of inducible nitric oxide synthase and COX-2 in macrophages in mice [9] and has been found to affect cellular control of ROS/RNS levels which can set in motion the DNA damage associated with tumorigenesis [9]. The therapeutic effect of CDDO results from its ability to upregulate Nrf2 by changing the conformation of the Nrf2-repressing, Kelch-like erythroid cell-derived protein with CNC homology-associated protein 1 (Keap1) [10]. Antioxidant response element (ARE) has been found by several animal and human studies to activate Nrf2-controlled antioxidant gene upstream [10]. Much research has been devoted to manipulating these compounds to generate new derivatives with higher sought after activities, increasing, for example, their anti-inflammatory activity and creating additional functional groups that could potentially be applied to the treatment of various disease states including kidney disease [10], obesity, and diabetes [11].

At present, CDDO derivatives have been used to treat lung injury [10], inflammation [12], and chronic kidney disease [13]. However, CDDO derivatives have not been found by preclinical trials to have equally positive results with GBM. Thus, if CDDO and CDDO derivatives are to be applied to this disease, much more research is needed to overcome their shortcomings. RTA 404, a trifluoroethylamide derivative of CDDO, has been found to have greater ability to cross the blood-brain barrier [14]. It has also been shown to enhance the Nrf2 expression and signaling in various models of neurodegeneration [15], including those that simulate multiple sclerosis [16], amyotrophic lateral sclerosis [17], and Huntington's disease [18]. It has also been found to induce apoptosis and prevent colony formation of Ewing's sarcoma [19] and neuroblastoma cells [19].

Although CDDO has been found to inhibit some cancers [20], it is unclear what effect RTA 404 may have on GBM and how it may achieve its effect. Because apoptosis and cell cycle regulation are often targets for cancer therapy, we studied the possible effects of RTA 404 on the delay of mitosis and gene expression in GBM 8401 and U-87-MG cells.

## 2. Materials and Methods

### 2.1. Materials

2-cyano-3,12-dioxo-N-(2,2,2-trifluoroethyl)-oleana-1,9(11)-dien-28-amide, RTA 404, was purchased from Cayman Chemical. DMSO (dimethyl sulfoxide) and MTT [3-(4,5-dimethylthiazol-2-yl)-2,5-diphenyltetrazolium bromide] were purchased from Sigma (St Louis, MO). Cell culture medium (DMEM), fetal bovine serum, antibiotics, sodium pyruvate, trypsin, and phosphate-buffered saline (PBS) were obtained from Gibco, BRL (Grand Island, NY). Polyvinylidene fluoride membrane (PVDF) (Millipore) and molecular weight markers purchased from Bio Rad (USA). All other reagents and compounds were of analytical grade.

### 2.2. Cell Culture

Human glioblastoma-astrocytoma U-87-MG (NCI-PBCF-HTB14; ATCC HTB-14) and human brain malignant glioma GBM 8401 cells were obtained from Bioresource Collection and Research Center (BCRC, Hsinchu, Taiwan) [21]. All cell lines were incubated in an atmosphere containing 5% CO_2_ at 37°C. GBM8401 cells were cultured in a RPMI1640 medium with supplemental 10% fetal bovine serum (FBS) and U87 MG cells in modified Eagle's Medium (MEM) with supplemental 10% FBS.

### 2.3. Cell Viability

A density of 3 × 10^4^ cells was suspended in culture medium containing 10% FBS and placed in a 96-well plate (0.1 ml of medium per each well) and incubated in an atmosphere containing 5% CO_2_, saturated humidity, and 37°C for 24 h. The cells were added with 0, 50, 100, 200, 400, or 500 nM RTA 404 and incubated with the 3-(4,5-dimethylthiazol-2-yl)-2,5-diphenyltetrazolium bromide (MTT) assay for 4 h. DMSO was added to stop the reaction, and optical density was determined at 540 nm with a multiwell plate reader (Powerwave XS, Biotek). When there were no cells, we subtracted the background absorbance of the medium.

### 2.4. Synergism between RTA 404 and TMZ on GBM 8401 and U87MG Cell Lines

The U87 MG and GBM8401 cells were placed in 6-well plates (1 × 10^6^) in a cell incubator and cultured overnight. The next day, the cells were treated with RTA 404 and cultured in a cell incubator for 48 hours. The cells were then washed with PBS, and 1× trypsin was added and placed in a 37°C oven for 1-2 minutes. After the cells had fallen naturally, a new DMEM culture medium was used to collect them into a 15 ml centrifuge tube. After they were evenly mixed, 10 *μ*l of the cell solution was added to the cell counter to determine the number of cells in each group. The cells were replaced into various media in 24-well plates (3 × 104) with various concentrations from TMZ (200-1000 uM) or without TMZ and cultured in a cell incubator for 72 hours. The cell survival rate was determined by MTT.

Data from the cell viability assay experiments were employed used to calculate the combination index (CI) using the following formula [22]: CI = D1/(D*x*)1 + D2/(D*x*)2, where D1 and D2 are the concentrations of reagents 1 and 2 applied in combination to achieve *x*% of the total drug effect, whereas (D*x*)1 and (D*x*)2 represent the concentrations of individual agents to accomplish the same efficacy. The CI value is a mathematical and quantitative representation of the pharmacological interplay of two drugs (CI > 1, antagonism; CI = 1, additive; CI < 1).

The dose-reduction index (DRI) was calculated by using following formula [22]: (DRI)1 = (D*x*)1/(D)1 and (DRI)2 = (D*x*)2/(D)2, where D1 and D2 are the concentrations of reagents 1 and 2 applied in combination to achieve *x*% of the total drug effect, whereas (D*x*)1 and (D*x*)2 represent the concentrations of individual agents to accomplish the same efficacy. A DRI value of more than 1 is of practical significance because it signifies therapeutic importance under circumstances in which the dose of any specific component of combinatorial drugs is reduced to ameliorate side effects caused by cytotoxicity to normal cells.

We calculated the values of CI and DRI at the 50% inhibition level, and (D*x*)1 and (D*x*)2 are the concentrations of RTA 404 and TMZ, respectively, that induce a 50% inhibition of cell growth; (D)1 and (D)2 are the concentrations of RTA 404 and TMZ in combination that also inhibits cell growth by 50%.

### 2.5. Synergism between RTA 404 and Radiation on GBM 8401 Cell Line

The GBM8401 was placed in a 6-well plate (1 × 10^6^) in a cell incubator and cultured overnight. The next day, the cells were treated with RTA 404 and cultured in a cell incubator for 48 hours. The cells were then washed with PBS, and they were added with 1× trypsin and placed in a 37°C oven for 1-2 minutes. After the cells had fallen naturally, a new DMEM culture medium was used to collect the cells into a 15 ml centrifuge tube. After the cells were evenly mixed, 10 *μ*l of the cell solution was added to the cell counter to determine the number of cells in each group. Different cell numbers were implanted according to different radiation intensities, 0 Gy: 100 cells, 1 Gy: 200 cells, 2 Gy: 400 cells, 4 Gy: 1000 cells, and 8 Gy: 10,000 cells. The seeded cells were irradiated under different intensities and placed in a cell incubator for seven days. The culture medium was changed every two days. After seven days, the cells were fixed with 37% formaldehyde for 15 minutes. After staining with 0.5% crystal violet, the cells were rinsed with water and washed with the crystal violet. The number of cell colonies that had formed was counted. The numbers were used in a formula calculating surviving fraction (SF). Plating efficiency was defined as the percentage of cells that had grown into colonies: PE = number of colonies counted/number of cells seeded and SF = number of colonies counted/number of cells seeded.

### 2.6. Cell Cycle Analysis

The U87 MG and GBM8401cells were placed in 6-well plates (1 × 10^6^) and placed in a cell incubator and cultured overnight. The next day, the cells were centrifuged in a 10 ml centrifuge tube, and the supernatant was collected. They were washed two times in PBS and added with 1× trypsin. They were then placed in a 37°C oven for 1-2 minutes. Once the cells fell off, they were collected in a centrifuge tube run at 2500 rpm for 5 minutes to remove the supernatant. One milliliter of PBS was added to wash the remaining culture solution, and the cells were centrifuged again at 2500 rpm for five minutes. 500 *μ*l of PBS was added to break up the cell pallet, and then 500 *μ*l of 70% ethanol was slowly added to the cells for fixation. They were placed in a refrigerator and left there overnight. The next day, those cells were centrifuged at 2500 rpm for 5 min, and the supernatant containing ethanol was removed. The cells were washed in 1 ml PBS. 5 *μ*l of RNAse A 100 mg/ml was added to PBS and placed in an oven at 37°C. After 30-minute reaction, 20 *μ*l of propidium iodide 2 mg/ml (final concentration 40 *μ*g/ml) was added, and the cells were placed in an oven at 37°C for 15 minutes. After that, the cells were transferred from the centrifuge tube to the Falcon tube. The sample was mounted on the machine (Beckman coulter FC500 and FACS Calibur, BD, USA). Data was analyzed using WinMDI 2.8 free software (BD, USA).

### 2.7. Migration Assay

U87 MG and GBM8401 cells were washed with PBS, they were added with 1× trypsin, and they were placed in a 37°C oven for 1-2 minutes. After the cells had naturally fallen off, fresh DMEM culture medium was used to collect the cells in a 15 ml centrifuge tube. 10 *μ*l of cell fluid was added to the cell counter which was used to count the number of cells of each cell in culture inserts (U87MG: 1 × 10^6^, GBM8401: 5 × 10^5^). They were placed in a cell culture incubator overnight. The next day, the culture inserts were placed under a microscope at 0 h and observed at 12, 24, and 48 hours.

### 2.8. Invasion Assay

Cell invasion assays were performed in vitro in Transwell chambers (COR3452; CORNING, Corning, NY, USA). To do this, cells were seeded at 5 × 10^5^ per insert, and 2 ml of medium was added to the lower chamber of each Transwell. After incubation for 24 h, cotton squabs were used to remove the cells remaining on the upper surfaces of the Transwell membranes. Cells that had made it through the membranes to the bottom of the insert were fixed, stained, and photographed. The numbers of cells in six random high-powered fields was counted.

### 2.9. Adhesion Assay

The U87 MG and GBM8401 cells were washed with PBS, they were added with 1× trypsin, and they were placed in 37°C oven for 1-2 minutes. Once the cells had slid down naturally, they were collected in a DMEM culture medium in a 15 ml centrifuge tube. 10 *μ*l of cell fluid was added to the cell counter, the numbers of cells of each cell in 24-well plate (3 × 10^4^) were counted, and then they were placed in the cell culture incubator. They were then fixed with 37% formaldehyde for 15 minutes, washed with PBS, stained with 0.5% crystal violet (Sigma-Aldrich; Louis, MO, USA), rinsed with water, washed the crystal violet, and observed and photographed under a microscope.

### 2.10. Senescence Assay

The senescence assay was performed using a Senescence *β*-Galactosidase Staining Kit (Cell Signaling, #9860). To do this, cells were seeded into 6-well plates in complete medium. Twenty-four hours later, the medium replaced with a RTA 404-supplemented medium. Seventy-two hours later, the cells were fixed and incubated with the *β*-galactosidase staining solution without CO_2_ in a dry incubator (37°C) overnight. The next day, the cells were studied under a microscope.

### 2.11. Western Blotting

All samples were lysed in 200 *μ*l of lysis buffer. A total of 50 *μ*g of protein per sample was loaded into the wells of a sodium dodecyl sulfate-polyacrylamide gel (SDS-PAGE) and subjected to electrophoresis at 50 V for 4 h. The separated proteins were subsequently transferred to PVDF membranes. After incubation for 1 h in blocking buffer, the membranes were incubated with primary antibodies [N-cadherin (1 : 1000; proteintech; 22018-1-AP), E-cadherin (1 : 1000; proteintech; 20874-1-AP), p-AKT (1 : 1000; proteintech; 22018-1-AP), AKT (1 : 1000; proteintech; 22018-1-AP), p21 (1 : 1000; Cell Signaling; #2947), and *β*-actin (1 : 20000; Sigma; A5441)] for 2 h at room temperature. Subsequently, the membranes were incubated with secondary antibodies (AP132P and AP124P; Millipore, Billerica, MA, USA) or a secondary antibody (IRDye; Li-COR, USA) for 90 min. Enhanced chemiluminescence solution (Western Lightning, 205-14621; Perkin Elmer, Waltham, MA, USA), a MiniChemi™ imaging and analysis system (Beijing Sage Creation, Beijing, China), and a near-infrared imaging system (Odyssey LI-COR, USA) were used to detect specific protein bands. Data were analyzed using Odyssey 2.1 software.

### 2.12. Data Analysis

A one-way analysis of variance (ANOVA) was used to compare proliferation, migration, and invasion assay results. A *p* value <0.05 was considered significant. Results are expressed as percentage of the control, control being 100%. All data are reported as the mean (± SEM) based on results of at least three separate experiments. All statistical operations were performed using SPSS 24.0 software (SPSS, Inc., Chicago, IL, USA).

## 3. Result

### 3.1. RTA 404 Inhibited Survival/Proliferation of GBM 8401 and U87 MG Cells

**Figure 1:**
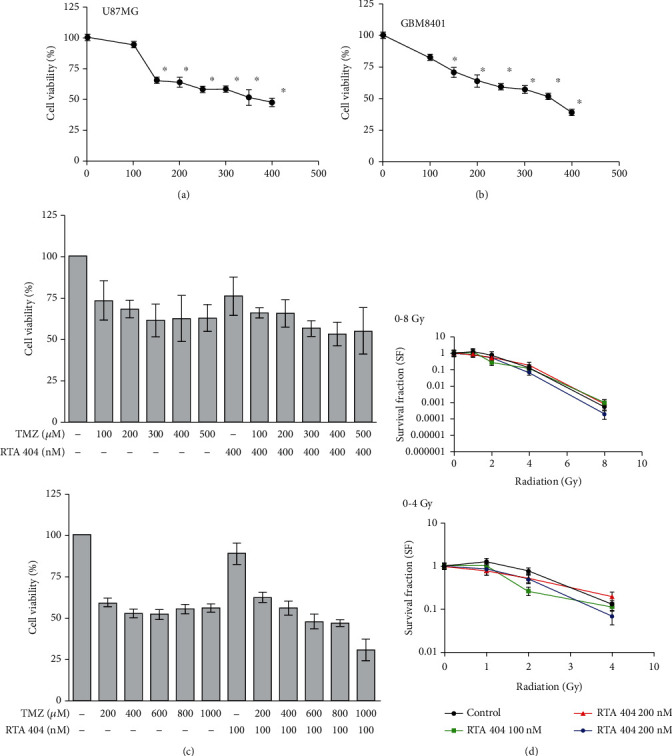
RTA 404 mediates the survival of GBM8401 and U-87-MG cells by inhibiting proliferation: (a) and (b) cells were treated with increasing doses of RTA 404 for 24 h in vitro. (c)The GBM cells were cotreated with increasing doses of RTA 404 with radiation in vitro. (d) The GBM cells were cotreated with increasing doses of RTA 404 with TMZ in vitro. The survival of RTA 404-treated cancer cells was measured using the MTT assay. Results are expressed as percentage of the control, control being 100%. Statistical analysis was performed using the *t*-test, with differences between the treatment and control groups (0 nM RTA 404) considered significant at *p* < 0.05, delineated by ^∗^.

We wanted to know whether RTA 404 could mediate the survival of human brain malignant glioma cells (GBM 8401 and U87 MG) and their proliferation. To find out, we performed an in vitro study in which we treated each of the glioma cell lines with increasing doses of RTA 404 (0, 100, 200, 300, and 400 nM) for 24 hours and then measured proliferation by MTT. As can be seen in Figure 1, while there was no change in normal skin fibroblast Hs-68 cells or normal lung fibroblast MRC-5 cells (data not shown), there was a significant dose-dependent decrease in survival and proliferation of the U87 MG and GBM 8401 cells (U87 MG; *y* = −0.0626*x* + 0.74, *R*^2^ = 0.9357, GBM8401; *y* = −0.1494*x* + 1.9791, *R*^2^ = 0.9067) 24 h after treatment. RTA 404 IC50 values were 321 and 786 for GBM8401 cells and U87 MG cells, respectively. Evaluating cytotoxicity response in GBM 8401 and U87 MG cells cotreated with different concentrations of RTA 404 and TMZ or radiation in vitro, we found a strong synergistic effect cell response cotreatment with either radiation or TMZ. RTA 404 and TMZ had synergistic effect on cytotoxicity (CI > 1 and DRI < 1), synergism corresponding to CI > 1 always yielding an unfavorable DRI < 1. RTA 404 CI50 values were 1.31 > 1 and 1.51 > 1 for GBM8401 cells and U87 MG cells, respectively. RTA 404 DRI50 values were 3.21 > 1 and 1.965 > 1 in GBM8401 cells and U87 MG cells, respectively. As shown in Figures 1(c) and 1(d), the results of our cell viability analysis by the MTT assay, growth was not significantly suppressed in cells cotreated with increasing doses of RTA 404 and either radiation or TMZ.

### 3.2. Cell Locomotion of Glioma Cell Lines Were Attenuated by RTA 404 Treatment

#### 3.2.1. RTA404 Attenuated Glioma Cell Migration

**Figure 2:**
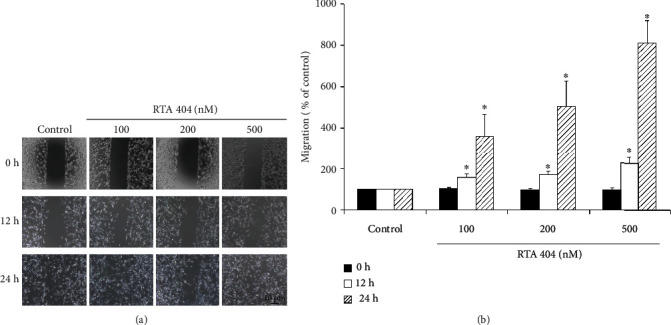
Influence of RTA 404 on migration ability of GBM cell lines. (a) Migration analysis of U87MG cells using different concentrations RTA 404 (100, 200, 500 nM). (b) RTA 404 reduced the migration ability of GBM cell lines. Results are expressed as percentage of the control, control being 100%. Statistical analysis was performed using the *t*-test, with differences between the treatment and control groups (0 nM RTA 404) considered significant at *p* < 0.05, delineated by ^∗^.

We were interested in what role RTA 404 might play in cell locomotion in GBM. To investigate, we performed a wound healing (migration) assay with glioma cells exposed to RTA 404. The wound healing assay was used to evaluate cell migration in the cell lines treated with different concentrations of RTA 404 (100, 200, and 500 nM). Cell migration was found to be in U87 MG (Figure 2(a)). In U87 MG cells, RTA 404 markedly inhibited the glioma cell migration at 0 hr, 12 hrs (100 nM, *p* < 0.001; 200 nM, *p* < 0.001; 500 nM; *p* < 0.001), and 24 hrs (100 nM, *p* < 0.001; 200 nM, *p* < 0.001; 500 nM; *p* < 0.001) (Figure 2(b)). Based on results depicted in Figure 2, RTA 404 may mediate the migration of GBM cell lines.

#### 3.2.2. RTA 404 Attenuated Glioma Cell Invasion

**Figure 3:**
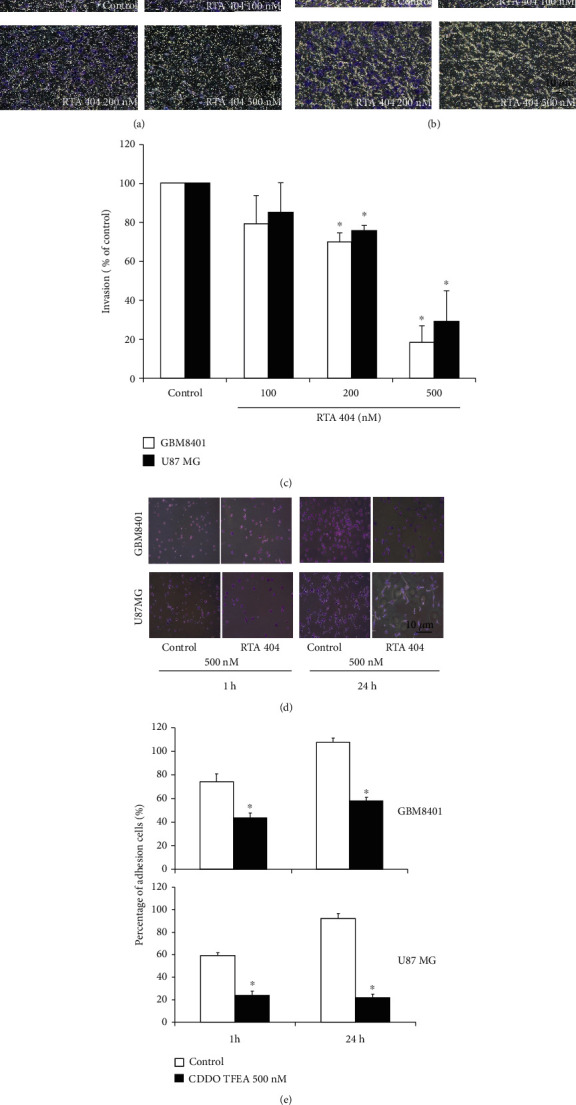
Influence of RTA 404 on invasion and adhesion ability of GBM cell lines. RTA 404 reduced the invasion ability of (a) U87 MG and (b) GBM8401 cells. (c) RTA 404 reduced the invasion ability of GBM cell lines. (d) and (e) The adhesion assay found marked inhibition of the cell lines treated with RTA 404 (500 nM) at 24 hours. RTA 404 reduced the adhesion of GBM cell lines. Statistical analysis used the *t*-test, with significance set at ^∗^*p* < 0.05 for the RTA 404 0 nM group.

We performed a Matrigel invasion assay of glioma cells exposed to RTA 404 (100, 200, and 500 nM). Cell invasion was suppressed in both U87 MG and GBM8401 cells (Figures 3(a) and 3(b)). RTA 404 simultaneously reduced percentage of cell invasion in both cell lines (U87 MG *y* = −22.322*x* + 128.23, *R*^2^ = 0.8768; GBM 8401 *y* = −25.378*x* + 130.42, *R*^2^ = 0.8949; *p* < 0.05 compared to cells not treated with RTA 404). As can be seen in Figure 3(b), RTA 404 markedly inhibited the invasive capability in both GBM8401 cells (100 nM, *p* = 0.034; 200 nM, *p* = 0.01; 500 nM; *p* < 0.001) and U87 MG cells (100 nM, *p* = 0.152; 200 nM, *p* = 0.111; 500 nM; *p* = 0.004).

#### 3.2.3. RTA 404 Attenuated Glioma Cell Adhesion

As can be seen in Figures 3(d) and 3(e), adhesion assays were performed on untreated control cell lines and RTA 404 treated cell lines. RTA 404 markedly inhibited GBM8410 cell adhesion at 1 hour (*p* = 0.002) and 24 hours (*p* < 0.001) as well as U87MG cell adhesion at 1 hour (*p* < 0.001) and 24 hours (*p* < 0.001). Inhibition was marked in both lines treated with RTA 404 (500 nM) at 24 hours.

#### 3.2.4. RTA 404-Induced Accumulation of the *G*_2_/*M* Phase U87 MG and GBM 8401 Cells

**Figure 4:**
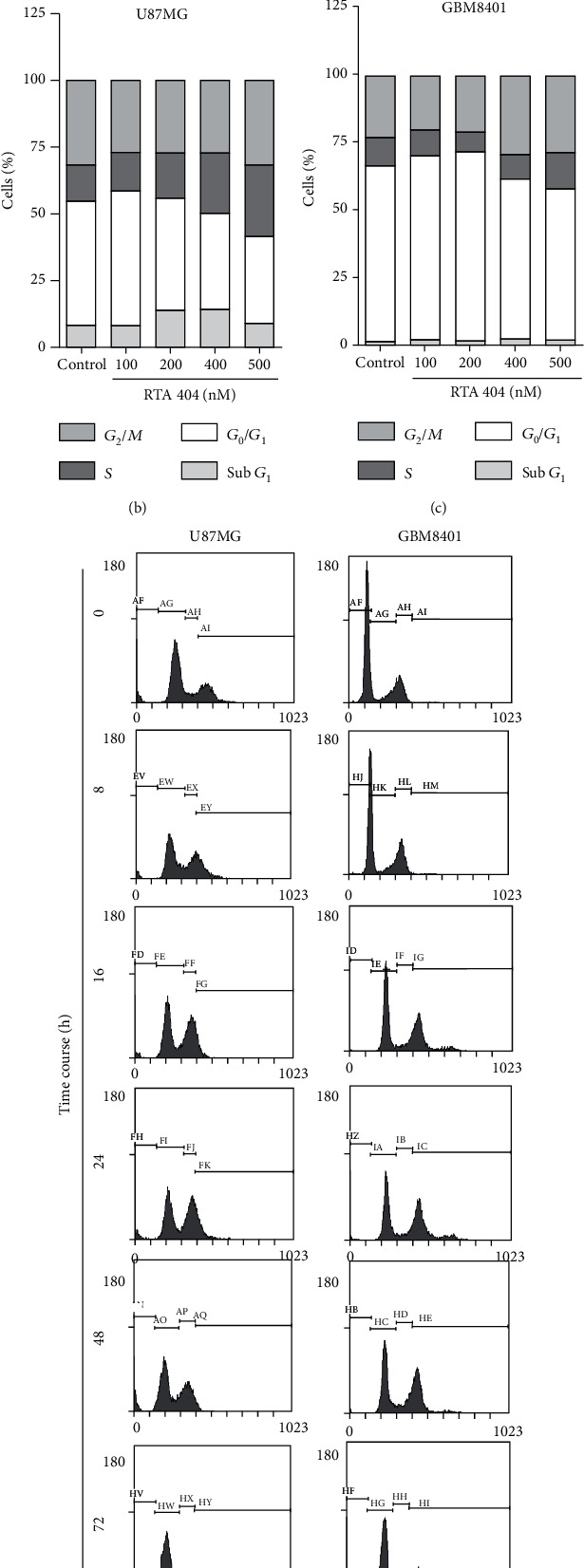
Effects of RTA 404 on cell cycle progression/distribution in GBM cell lines. (a, d) Cell cycle analysis of the cancer cells after being cultured with RTA 404. (b, c, e, and f) RTA 404 increased *S* and *G*_2_/*M* phase cell percentages (%). Cells were stained with propidium iodide to analyze the DNA content, quantified by flow cytometry.

The cell cycle distribution of RTA 404-treated cells was analyzed using flow cytometry. Cells were exposed to RTA 404 at different concentrations (0, 100, 200, 400, 500 nM) (Figure 4(a)) and different lengths of time (0 h, 8 h, 18 h, 24 h, 48 h, 72 h) (Figure 4(d)). Exposure to RTA 404 resulted in a dose-dependent increase in the number of cells in the *S* phase and *G*_2_/*M* phase, suggesting a reduction in mitosis in U87 MG and GBM 8401 cells, though cell cycle did not change over time. Furthermore, RTA 404 increased the number of cell populations in the *S* phase (Figures 4(c) and 4(e)) and *G*_2_/M phase (Figures 4(b) and 4(f)) while simultaneously reducing the number of cells in the *G*_1_ phase (Figures 4(c)–(f)).

#### 3.2.5. RTA 404-Induced Senescence Was in Glioma Cell Lines

**Figure 5:**
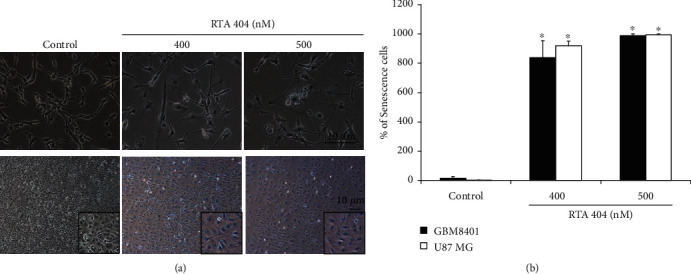
Effects of cell senescence in RTA 404-treated cells were analyzed using (a) *β*-galactosidase staining. (b) Results are expressed as a percentage of the control, control being 100%. Statistical analysis was performed using the *t*-test, with differences between the treatment and control groups (0 nM RTA 404) considered significant at *p* < 0.05, delineated by ^∗^.

Cell senescence was analyzed using *β*-galactosidase staining.  Figure 5(a) shows cells exposed to RTA 404 at different concentrations (0, 400, and 500 nM) before processing and analysis.  Figure 5(b) shows that exposure to RTA 404 increases the number of senescent cells. In both GBM8401 cells and U87 cells, RTA 404 increased senescence at 400 nM (both *p* < 0.001) and 500 nM (both *p* < 0.001).

#### 3.2.6. RTA 404 Induced Glioma Cell Cycle Arrest and Apoptosis through the p21/AKT Signaling Pathway

**Figure 6:**
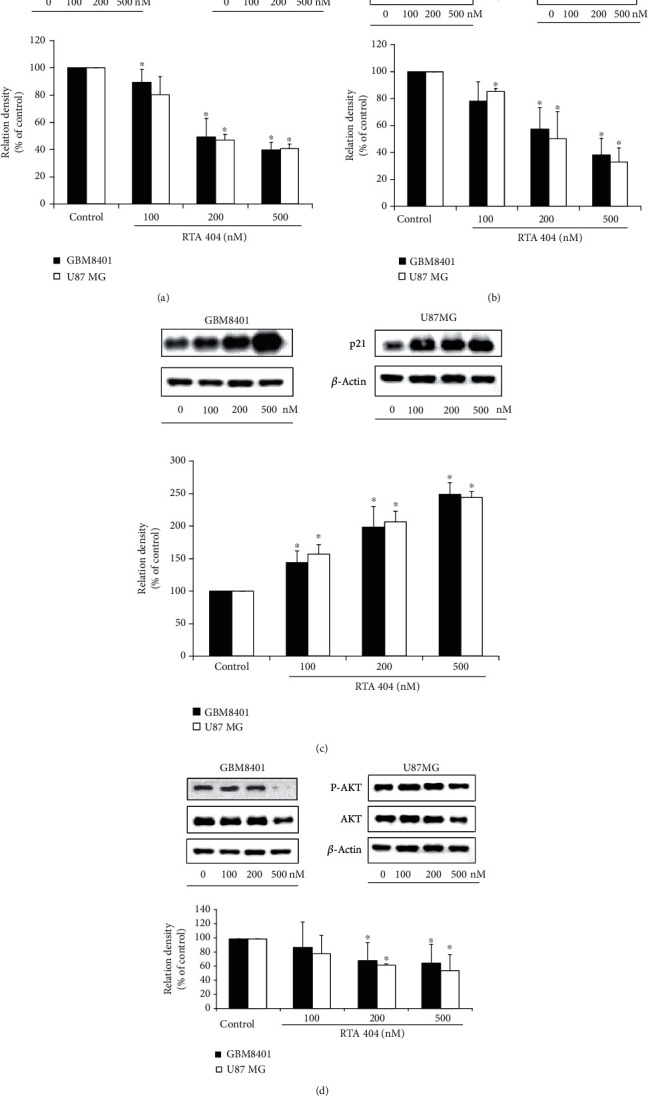
RTA 404 regulated the gene expression (N-cadherin, E-cadherin, p21, p-AKT, AKT) in GBM8401 and U-87-MG cells. Cells were treated with RTA 404 for 24 h. Gene and protein expressions were subsequently detected using Western blot analysis. Results are expressed as percentage of the control, control being 100%. Statistical analysis was performed using the *t*-test, with differences between the treatment and control groups (0 nM RTA 404) considered significant at *p* < 0.05, delineated by ^∗^.

As seen above, RTA 404 attenuated GBM cell proliferation and inhibited cell locomotion. To explore the mechanism through which it achieved these effects, we performed Western blot analysis of cellular proteins extracted from the brain cancer cell lines treated with RTA 404 to compare the relative intensities of N-cadherin, E-cadherin, p-AKT, AKT, and p21 gene expression (Figure 6). In GBM8401 cells, we found the protein expressions of N-cadherin (100 nM, *p* = 0.030; 200 nM, *p* = 0.004; 500 nM, *p* = 0.023), E-cadherin (100 nM, *p* = 0.006; 200 nM, *p* = 0.015; 500 nM, *p* = 0.020), and p-AKT/AKT (100 nM, *p* = 0.2494; 200 nM, *p* = 0.0395; 500 nM, *p* = 0.029) to be significantly downregulated and tumor suppressor gene p21 upregulated (100 nM, *p* = 0.002; 200 nM, *p* = 0.002; 500 nM, *p* = 0.006) in those treated with RTA 404. In U-87-MG cells, we found the protein expressions of N-cadherin (100 nM, *p* = 0.072; 200 nM, *p* = 0.051; 500 nM, *p* = 0.036), E-cadherin(100 nM, *p* = 0.080; 200 nM, *p* = 0.009; 500 nM, *p* = 0.040), and p-AKT/AKT (100 nM, *p* = 0.1476; 200 nM, *p* < 0.001; 500 nM, *p* = 0.0359) to be significantly downregulated and tumor suppressor gene p21 upregulated (100 nM, *p* = 0.007; 200 nM, *p* = 0.002; 500 nM, *p* = 0.005) in those treated with RTA 404. Because AKT has previously been targeted in attempts to control tumorigenesis (REF), we wanted to investigate whether RTA 404 exerted its effects through this pathway. We found that GBM cell lines treated with 500 nM RTA 404 had significant decreases in P-AKT, AKT, and P-AKT/AKT. These results suggest RTA 404 induced increases in senescence, *G*_2_/*M* phase arrest, and apoptosis may have occurred via the AKT/p21 signaling pathway.

## 4. Discussion

Triterpenoids, found in some plants including chrysanthemums, have been used in traditional herbal medicine in Asia. They have been synthesized into a large family of compounds through the cyclization of squalene [23]. Oleanolic acid (OA) and ursolic acid, both naturally occurring triterpenoids, have been found to have anti-inflammatory and anticarcinogenic activities, though weak [12]. A series of novel derivatives of OA have been synthesized to enhance their effectiveness [24]. One oleanane triterpenoid, known as 2-cyano-3-,12-dioxoolean-1,9-dien-28-oic acid (CDDO), is an Nrf2 activator found to play a role in cellular control of ROS/RNS levels associated with tumorigenic DNA damage [25]. CDDO was developed to reduce tumor cell proliferation [26]. Some of its derivatives, including CDDO methyl ester (CDDO-Me) and CDDO imidazolide (CDDO-Im), have antitumor effects [27]. The one we investigated in this study, RTA 404, has been found to have enhanced ability to cross the blood-brain barrier [18] and has been used an Nrf2 activator in the treatment of cancer. RTA404 has also been found to have neuroprotective effects in some animal models of degenerative diseases, including ischemic stroke [14] and autoimmune encephalomyelitis [16]. The administration of RTA 404 has also been found to significantly reduce neurological dysfunction and ischemic brain damage after middle cerebral artery occlusion in mice [14]. It has been found to protect nerves from damage by activating the Nrf2/ARE signaling pathway leading to reduced oxidative stress, improved motor impairment, and increased longevity in a transgenic mouse model [28].

This study found that RTA404 reduced glioma cell proliferation and attenuated glioma cell locomotion in GBM8401 and U87 MG cells. In addition, cell cycle analysis also showed that RTA 404 arrested glioma cells at the *S* and *G*_2_/*M* phase (Figure 4). These results suggest that RTA404 exerts its anticancer effects on glioblastoma via its effect on cell cycle. One previous cell cycle study showed RTA 404 brought about a decrease in the *S* phase and increases in sub-*G*_1_/*G*_0_ in human neuroblastoma cells, changes suggesting DNA degradation and apoptosis in these cells [19]. In the current study, we found RTA 404 decreased in the *G*_1_/*G*_0_ phase and increased in the *S* phase and *G*_2_/*M* phase, since cells trapped in the *G*_2_/*M* at the time RTA404 treatment did not progress into the *G*_1_/*G*_0_ phase.

Although current research indicates that triterpenoid derivatives affect cells through several pathways including NF*κ*B, Jak/STAT, and Nrf2-Keap1 signaling pathways [29], CDDO is an inhibitor of I*κ*B kinase (IKK) *β* which interferes with nuclear factor-*κ*B binding to DNA and downstream transcriptional activation [30]. It can also suppress STAT signaling associated with control of inflammation and cell proliferation [31]. Both CDDO and RTA 404 also have the ability to directly block IKK*β* activity and the NF-*κ*B pathway [32]. In current study, we found that RTA 404 dose-dependently inhibited the expression of p-AKT and increased the expression of p21. And after treatment of glioma cells with RTA 404 in this study, there was a decrease in the *G*_1_/*G*_0_ phase and increase in the *S* phase and *G*_2_/*M* phase, since the cells which were trapped in the *G*_2_/*M* at the time of treatment did not progress into the *G*_1_/*G*_0_ phase. Our results suggest that RTA 404 reduces the activation of the AKT pathway, which would otherwise protect cancer cells. The AKT pathway is known to affect angiogenesis, tumor proliferation, and metastasis via its regulation of VEGF and N-cadherin (Figure 6).

The antiproliferative effect of the senescence response makes it good therapeutic tumor suppressing target [33]. This response is mediated by ARF/p53 and INK4a/RB, two major tumor suppressor pathways [33]. Although most of the research on senescence has been performed in the context of nonmalignant primary cells, there is evidence suggesting that cancer cells can also enter senescence [34]. The challenge is to induce senescence in cancer cells and identify ways to selectively kill the senescent cancer cells [35]. As shown in Figure 6, RTA 404 induced cell entry to senescence and that loss through consecutive cell division limited the proliferation of GBM cells. These findings indicated RTA 404 induced the number of senescent GBM cells inhibiting their ability to proliferate. Based on our findings, we believe that RTA 404 arrest of glioma cell cycle may result from its effect on the p21/AKT signaling pathway.

Treatment with RTA404 produced consistent results for both GBM 8401 and U87 MG cell lines. RTA 404 markedly inhibited proliferation, invasion, and adhesion and increased the number of senescent in both GBM8401 cells and U87 MG cells, possibly occurring as a result of its effect on the p21/AKT signaling pathway. Although RTA404 produced consistent results for both cell lines, there were subtle differences in its effects on the two cell lines. For example, while RTA 404 inhibited the proliferation of GBM 8401 and U87 MG cells, their IC50 values differed 321 in GBM8401 cells and 786 in U87 MG cells, suggesting that RTA 404 had a greater inhibitory effect on this higher malignant cell line. Therefore, there are some subtle differences in the treatment of the two cell lines, but they will not affect the overall effect of RTA 404 on the two cell lines.

## 5. Conclusions

In summary, RTA 404 attenuated proliferation and inhibited GBM 8401 and U87 MG cell locomotion in vitro; it induced glioma cell cycle arrest and accumulation of the *G*_2_/*M* phase through the AKT/p21signaling pathway. Furthermore, it induced senescence in glioma cell lines. These findings suggest that RTA404 can inhibit proliferation, cell locomotion, cell cycle progression, and induce senescence in GBM cells in vitro, possibly though its inhibition of N-cadherin and E-cadherin expression via its inhibition of the AKT pathway.

## Data Availability

The data used to support the findings of this study are available in the article supplementary material.

## Abbreviations

ARE: Antioxidant response element
CDDO: 2-Cyano-3-,12-dioxoolean-1,9-dien-28-oic acid
CDDO-Im: CDDO imidazolide
CDDO-Me: CDDO methyl ester
CDDO-TFEA: CDDO trifluoroethyl amide
GBM: Glioblastoma multiforme
Keap1: Kelch-like ECH-associated protein 1
Nrf2: Nuclear factor erythroid 2-related factor 2
Oleanolic acid [36]
ROS: Reactive oxygen species
RNS: Reactive nitrogen species
WHO: World Health Organization.

## Supplementary Materials

Supplementary Materials Antibody: N-cadherin (1 : 1000; proteintech; 22018-1-AP). E-cadherin (1 : 1000; proteintech; 20874-1-AP). p-AKT (1 : 1000; proteintech; 22018-1-AP). AKT (1 : 1000; proteintech; 22. p21 (1 : 1000; Cell Signaling; #2947). *β*-Actin (1 : 20000; Sigma; A5441). (2) Image assay software: ImageJ and NIH. (3) Analysis software: SPSS. Figures 1(a) and 1(b): cell viability. Figure 1(c): cotreated with TMZ. Figure 1(d): cotreated with radiation. Figures 2(a) and 2(b): migration. Figures 3(a)–(c): invasion. Figures 3(d) and 3(e): adhesion. Figure (d): AK. Figures 5 (and 5 (b): senescence. Figure 6: Western blot. Figure 6(a): N-cadherin. Figure 6(b): E-cadherin. Figure 6(c): p21. Figure 6(d): p-AKT.
