# Supplementary material for: Therapeutic Potential of RTA 404 in Human Brain Malignant Glioma Cell Lines via Cell Cycle Arrest via p21/AKT Signaling
Source: Biomed Res Int. 2021 Mar 8;2021:5552226. doi: 10.1155/2021/5552226 (PMC7963900; doi:10.1155/2021/5552226)
Supplement: Supplementary Materials — Antibody: N-cadherin (1 : 1000; proteintech; 22018-1-AP). E-cadherin (1 : 1000; proteintech; 20874-1-AP). p-AKT (1 : 1000; proteintech; 22018-1-AP). AKT (1 : 1000; proteintech; 22. p21 (1 : 1000; Cell Signaling; #2947). β-Actin (1 : 20000; Sigma; A5441). (2) Image assay software: ImageJ and NIH. (3) Analysis software: SPSS. Figures 1(a) and 1(b): cell viability. Figure 1(c): cotreated with TMZ. Figure 1(d): cotreated with radiation. Figures 2(a) and 2(b): migration. Figures 3(a)–(c): invasion. Figures 3(d) and 3(e): adhesion. Figure (d): AK. Figures 5 (and 5 (b): senescence. Figure 6: Western blot. Figure 6(a): N-cadherin. Figure 6(b): E-cadherin. Figure 6(c): p21. Figure 6(d): p-AKT. [file 5552226.f1.pdf]

Supplement data

1. Antibody:

N-cadherin (1:1000; proteintech; 22018-1-AP)
E-cadherin (1:1000; proteintech; 20874-1-AP)
p-AKT (1:1000; proteintech; 22018-1-AP)
AKT (1:1000; proteintech; 22018-1-AP)
p21 (1:1000; Cell Signaling; #2947)
β-actin (1:20000; Sigma; A5441)

2. Image assay software: ImageJ, NIH.

3. Analysis software: SPSS

Supplement data

Fig6(A).N-Cadherin

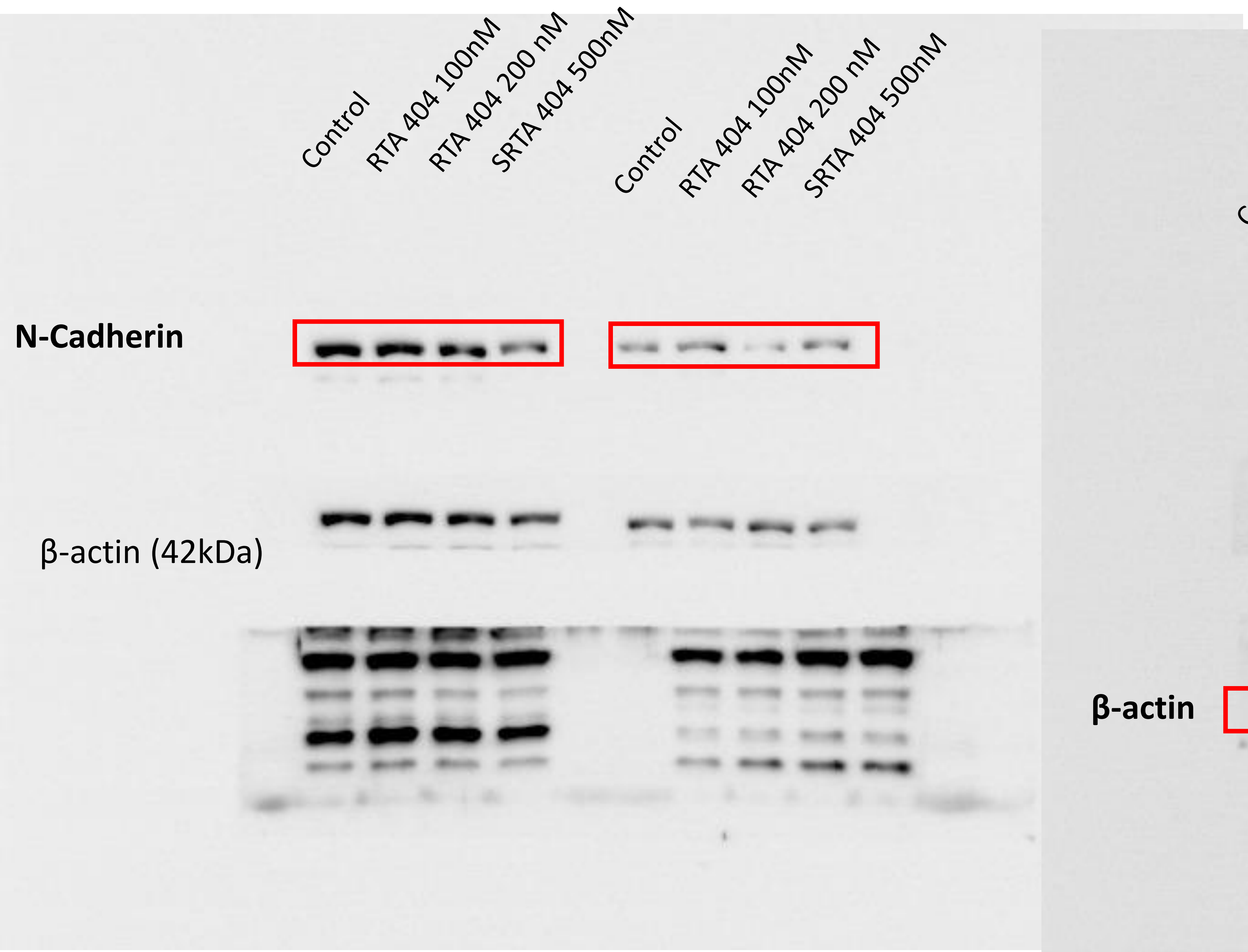

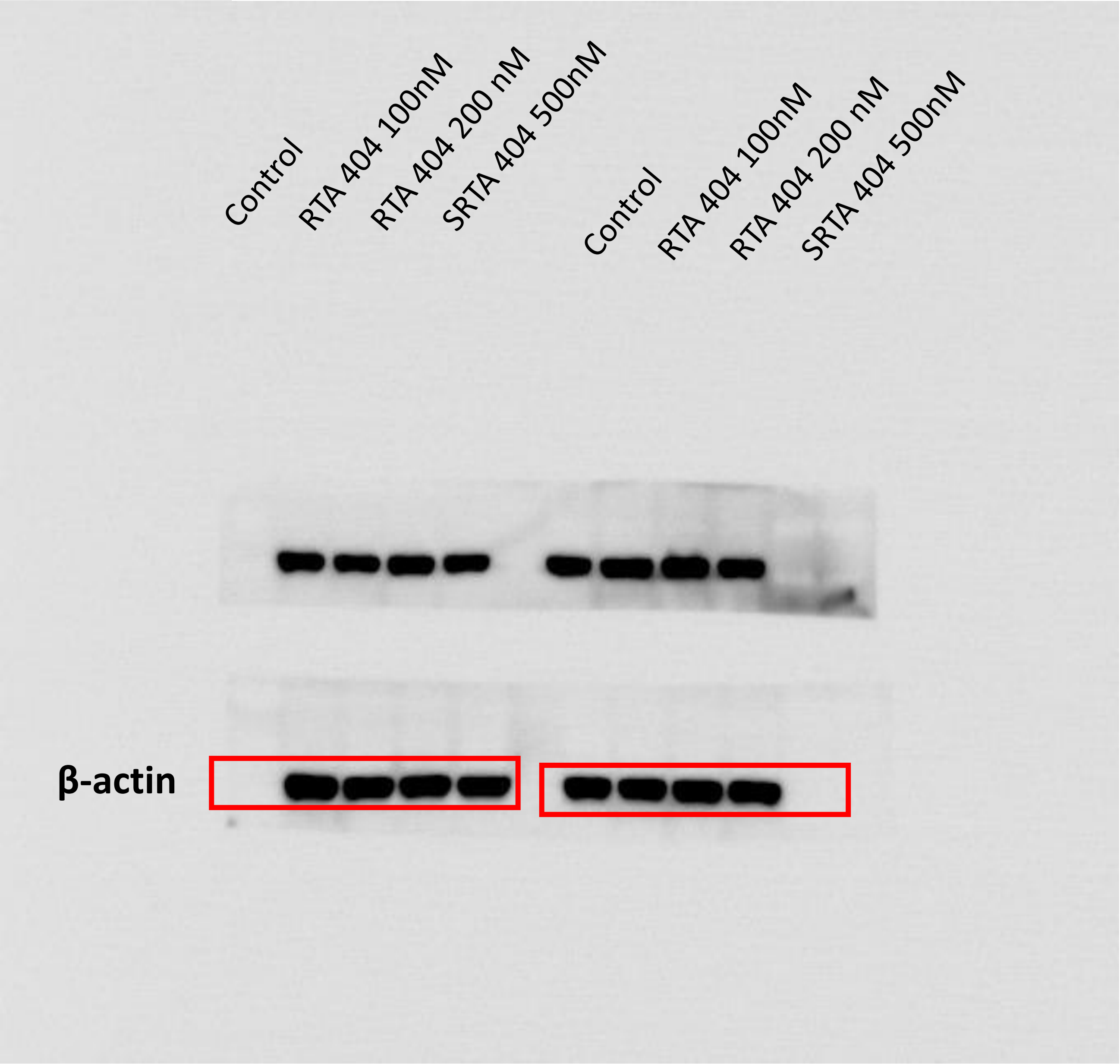

Supplement data

Fig6(B).E-Cadherin

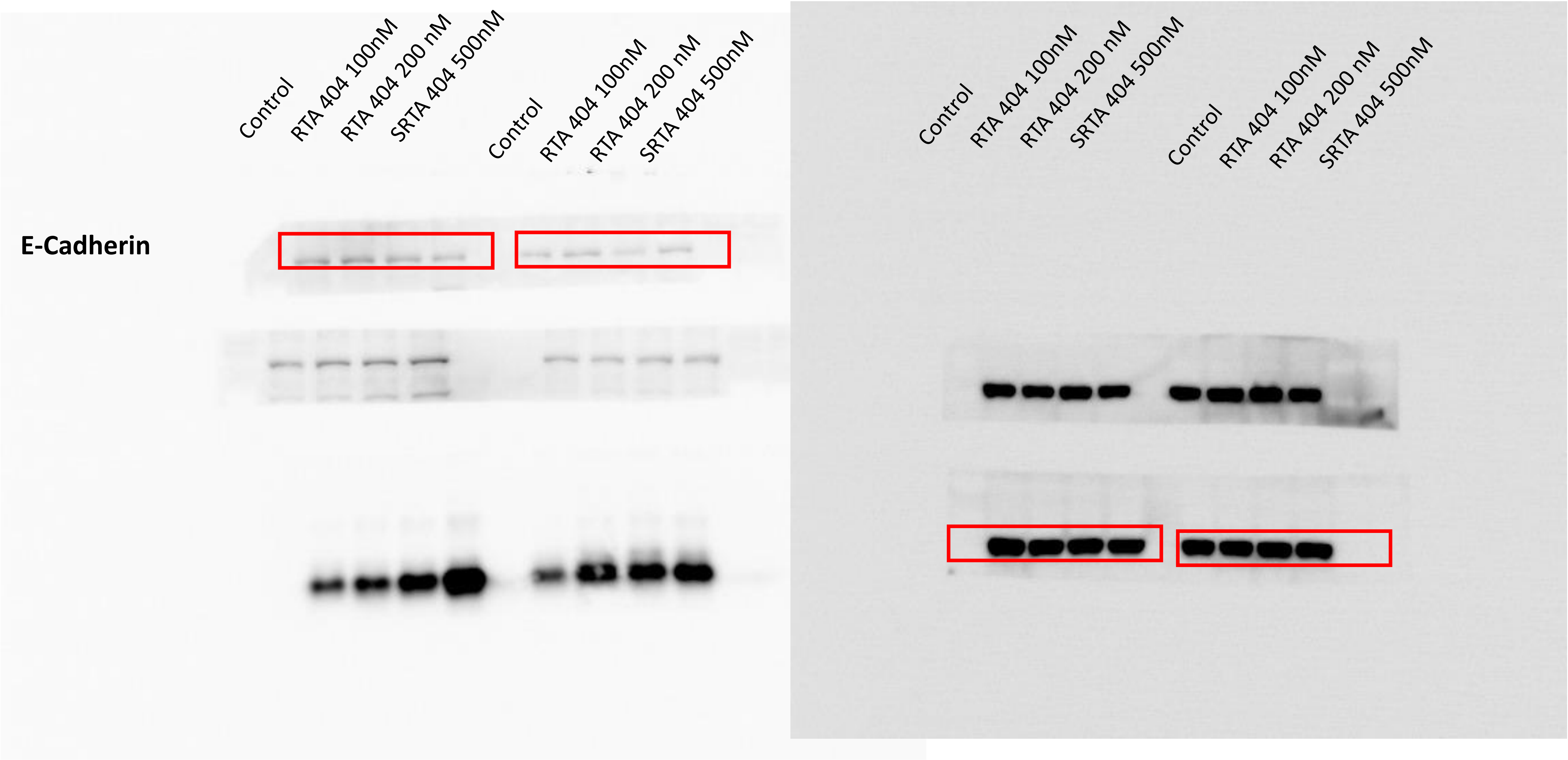

Supplement data

Fig6(C).p21

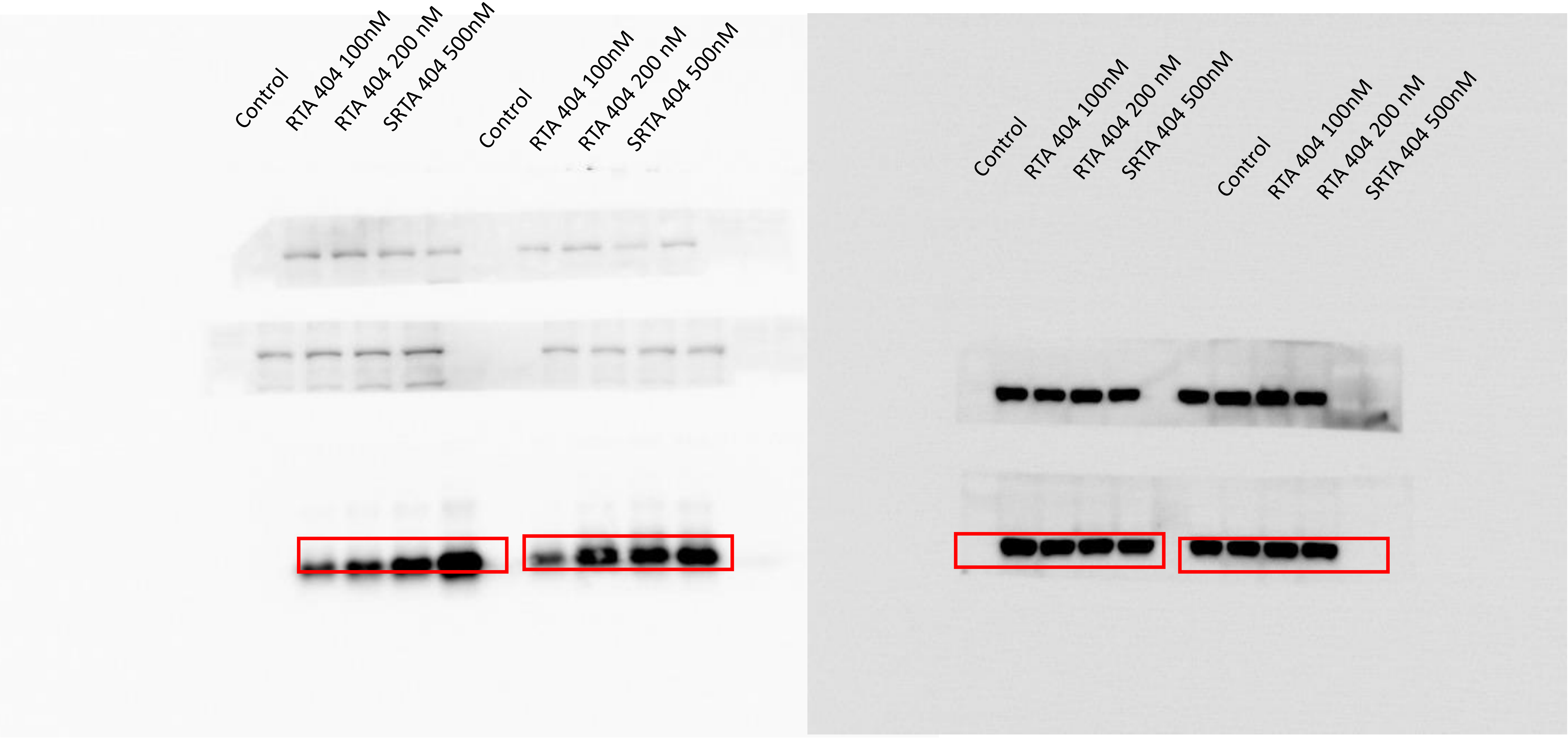

Supplement data

Fig(D)AKT

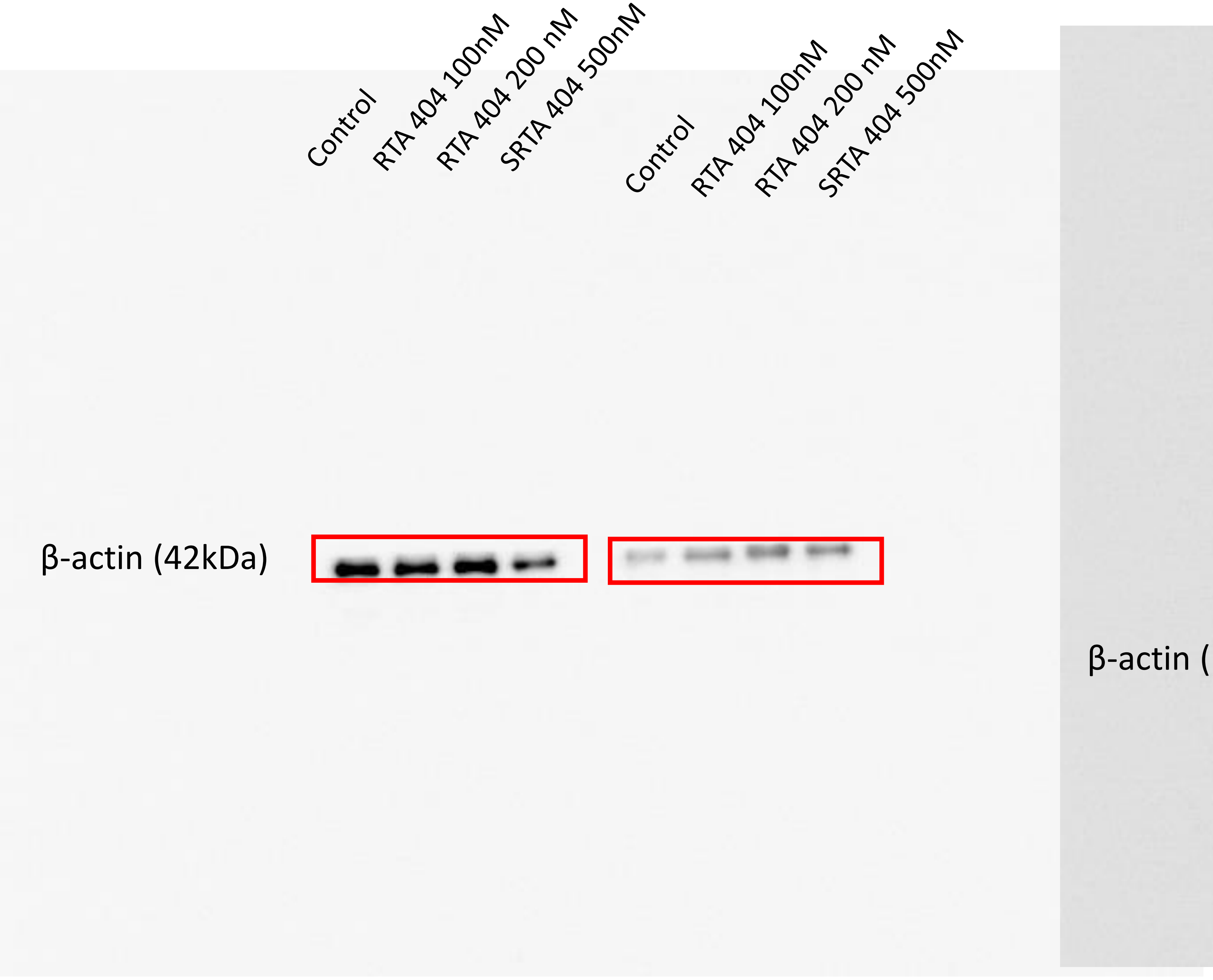

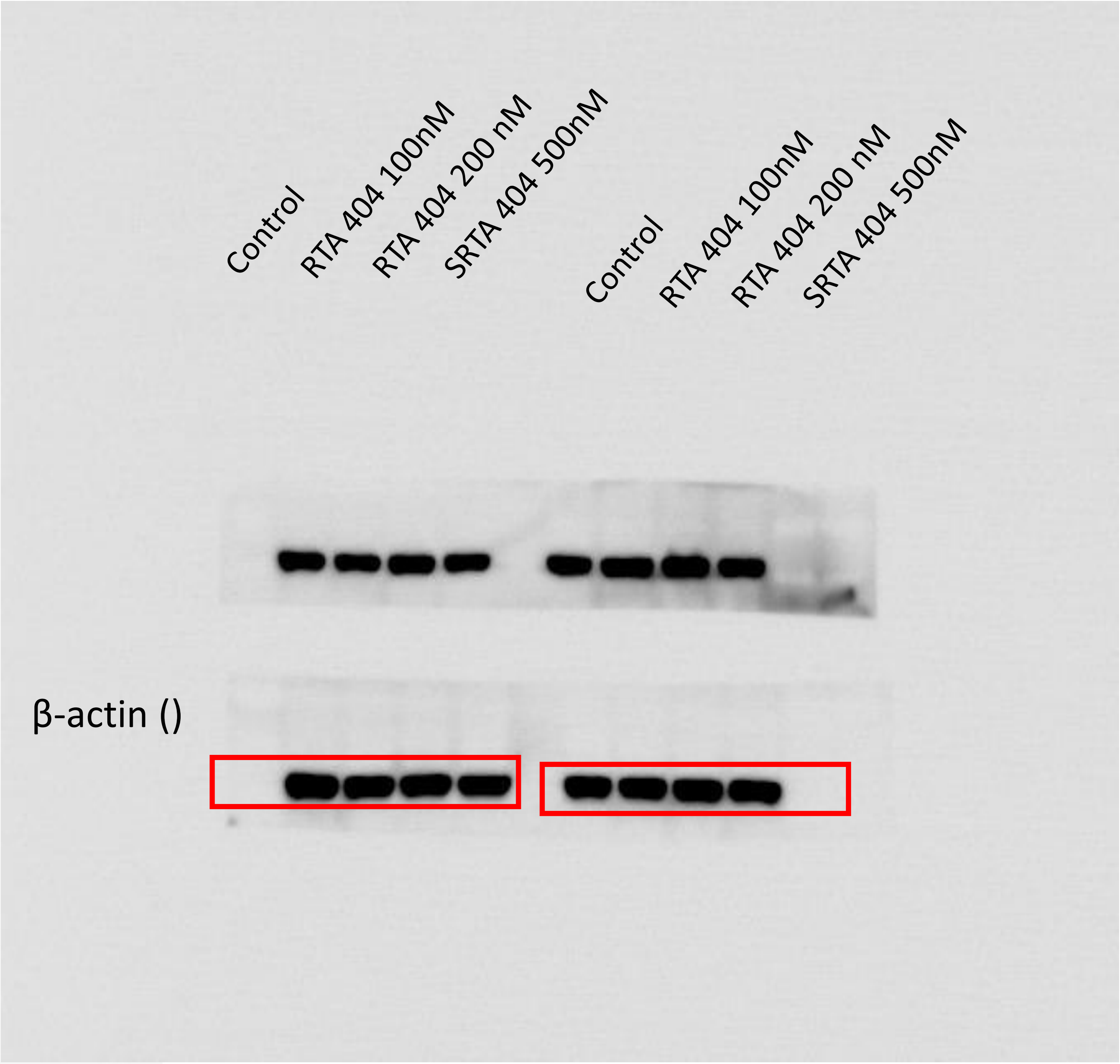

Supplement data

Fig6(D)p-AKT

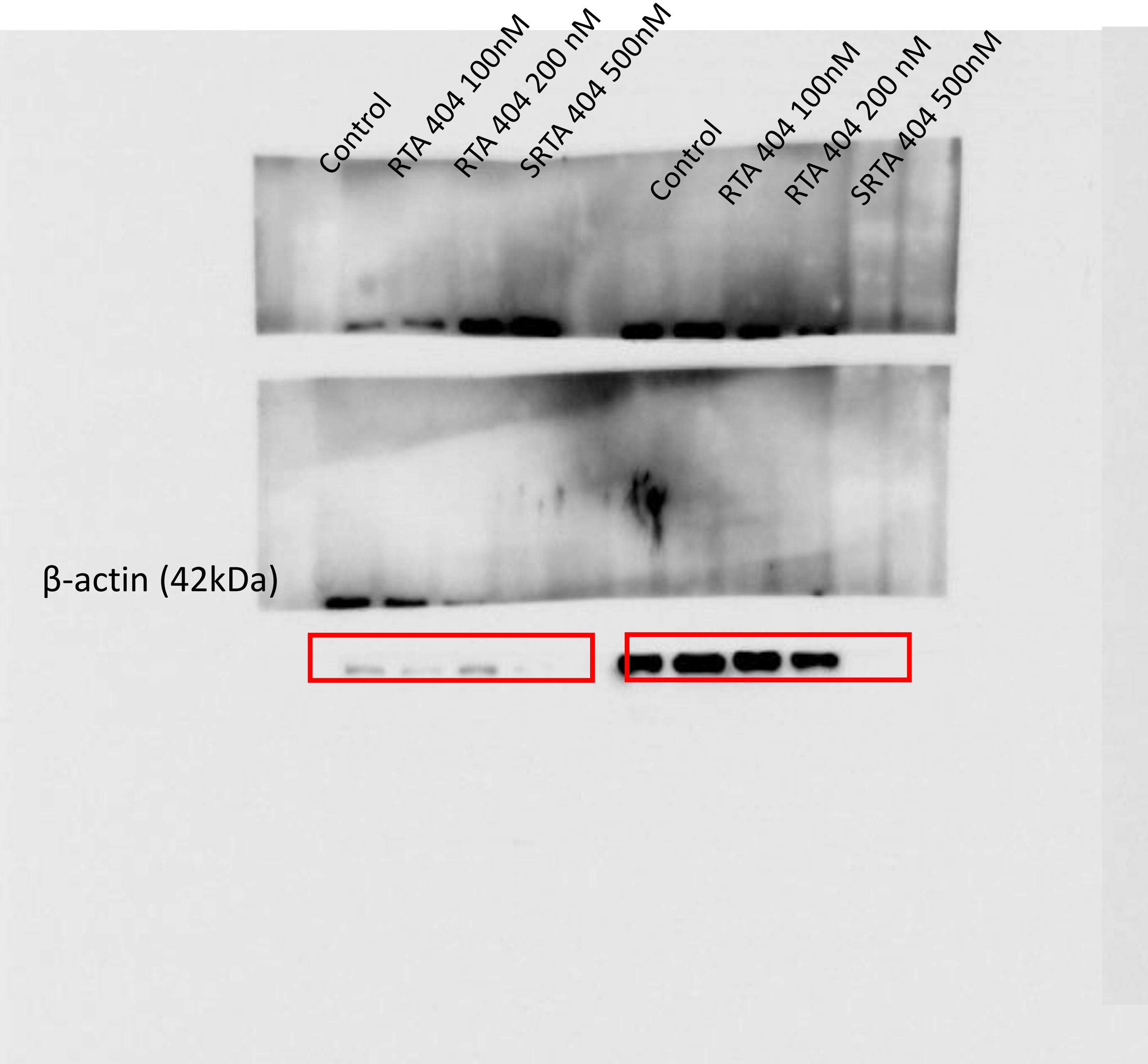

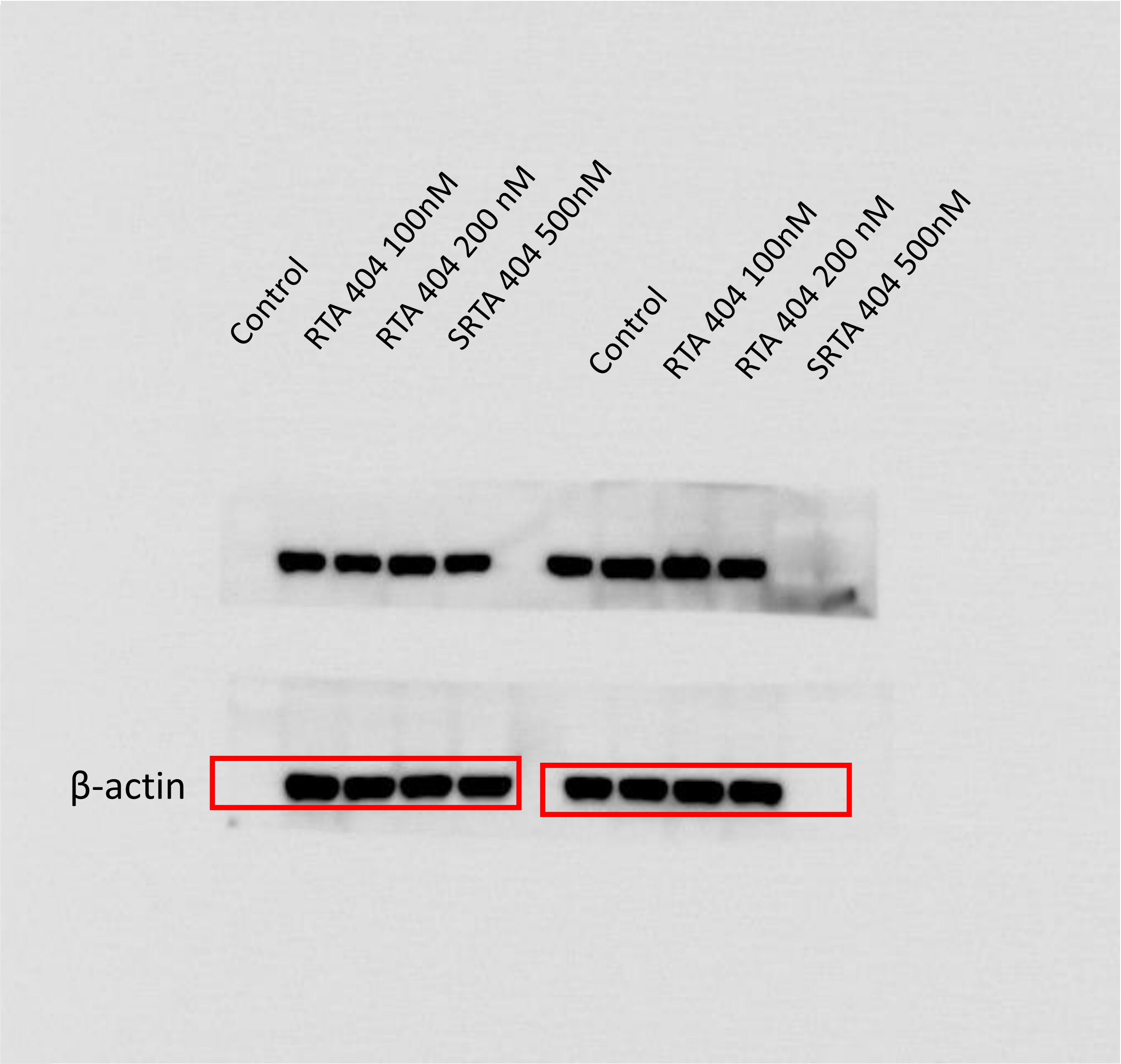

Supplement data

Raw data

Figure 1(A)(B) Cell viabiltiy

GBM8401							
Control	1	10	25	50	100	300	500
1.685	1.483	1.321	1.431	1.439	1.343	1.111	0.748
1.639	1.507	1.336	1.374	1.466	1.331	1.067	0.763
1.678	1.579	1.352	1.427	1.561	1.336	1.081	0.749
1.673	1.595	1.415	1.441	1.533	1.339	1.072	0.759
1.654	1.462	1.406	1.41	1.481	1.531	1.132	0.735
1.673	1.461	1.381	1.431	1.471	1.468	1.151	0.738
1.649	1.482	1.513	1.388	1.441	1.359	1.228	0.963
1.718	1.476	1.478	1.401	1.458	1.337	1.213	0.907

U87MG							
0	1	10	25	50	100	300	500
1.192	0.981	1.04	0.996	1.107	1.095	0.597	0.434
1.172	0.971	1.03	1.011	1.088	1.09	0.589	0.422
1.168	0.946	0.778	0.935	0.95	1.059	0.514	0.341
1.171	0.926	0.901	0.917	0.949	1.043	0.522	0.334
1.058	0.929	0.82	0.965	1.022	1.116	0.487	0.358
1.068	0.89	0.909	0.982	1.017	1.139	0.481	0.351
1.273	1.157	1.179	1.177	1.225	1.182	0.65	0.451
1.301	1.179	1.197	1.178	1.248	1.178	0.62	0.453

Supplement data

Raw data

Figure 1(C) Cotreated with TMZ

Co-treated with TMZ												
							RTA 404	100	100	100	100	100
	Con.	200	400	600	800	1000	100	200	400	600	800	1000
A	0.718	0.411	0.363	0.397	0.367	0.385	0.585	0.447	0.405	0.379	0.451	0.26
B	0.701	0.41	0.366	0.385	0.361	0.398	0.61	0.461	0.424	0.373	0.471	0.224
C	0.666	0.368	0.367	0.357	0.379	0.342	0.605	0.424	0.387	0.344	0.302	0.252
D	0.653	0.381	0.376	0.348	0.381	0.357	0.676	0.427	0.397	0.309	0.3	0.255
E	0.679	0.399	0.349	0.344	0.318	0.381	0.608	0.416	0.377	0.306	0.324	0.171
F	0.665	0.403	0.343	0.345	0.317	0.383	0.614	0.418	0.37	0.302	0.316	0.17
G	0.758	0.491	0.398	0.368	0.347	0.446	0.629	0.45	0.381	0.327	0.377	0.168
H	0.77	0.46	0.393	0.374	0.345	0.454	0.638	0.448	0.386	0.33	0.372	0.215

Supplement data

Raw data

Figure 1(D) Cotreated with radiation

0 Gy				1 Gy				2 Gy			
Control	45	43	42	Control	88	66	60	Control	65	60	50
150	50	48	54	150	88	84	91	150	55	61	50
200	34	55	51	200	41	62	68	200	53	47	66
400	16	11	14	400	23	11	28	400	13	17	13

4 Gy				8 Gy			
Control	46	44	39	Control	10	6	7
150	47	50	40	150	8	10	12
200	42	45	40	200	3	8	6
400	6	6	7	400	0	0	0

Supplement data

Raw data

Figure 2(A)(B) migration

Migrtion												
U87MG	0hr				12hr				24hr			
	Control	100nM	200nM	500nM	Control	100nM	200nM	500nM	Control	100nM	200nM	500nM
	4964691	4843065	4823152	4445215	1953170	2905839	2942122	4045145	490115	581167	1832797	3511718
	3799600	4033651	3923125	3861335	1595832	2379854	2785418	3436588	417956	1573123	2236181	3282134
	3750800	3957065	3543594	3873415	1312780	2295097	2409643	3408605	337557	1543255	2055284	3137466

Supplement data

Raw data
Figure 3(A)(B)(C) Invasion

GBM8401				U87MG		
Control	284	337	317	113	98	82
CDDO-TFEA 100uM	266	218	253	79	84	82
CDDO-TFEA 200uM	194	224	238	88	71	63
CDDO-TFEA 500uM	34	93	51	13	41	27

Supplement data

Raw data:

Figure 3(D)(E)Adhesion

GBM 8401						
	1h			24h		
Control	74	68	81	104	112	107
CDDO-TFEA 500nM	47	44	40	58	61	55

U87MG						
	1h			24h		
Control	57	62	59	89	96	92
CDDO-TFEA 500nM	29	22	21	22	19	25

Supplement data

Raw data:

Figure 5(A) (B)sensence

GBM8401				U87-MG			
Control	20	20	0	Control	0	4	1
CDDO-TFEA 400uM	710	890	920	CDDO-TFEA 400uM	892	914	953
CDDO-TFEA 500uM	990	1000	980	CDDO-TFEA 500uM	994	991	1000

Supplement data

Raw data:

Figure 6 Western blot

Western Blot

GBM8401				U87MG			
**N-Cadherin**							
C	100	200	500	C	100	200	500
4415	4185	2667	1881	2307	2173	1138	978
3857	3656	2058	1298	6202	4879	2595	2642
2016	1576	685	873	3241	2198	1599	1195
**p-21**							
C	100	200	500	C	100	200	500
7768	9951	13487	17682	6235	9402	12697	14543
5173	7290	9700	13541	8317	12253	15885	20446
4154	6761	9723	10653	6676	11575	14953	16816
**E-Cadherin**							
C	100	200	500	C	100	200	500
6683	5995	4892	3408	2637	2280	1087	794
5839	4819	3334	2144	9388	7748	6872	4165
2304	1434	949	603	3705	3203	1343	881
**p-AKT**							
C	100	200	500	C	100	200	500
896	392	793	144	6434	6008	3309	2333
596	528	465	75	959	348	586	154
4286	3320	1520	1553	6891	4631	3543	2173
**AKT**							
C	100	200	500	C	100	200	500
7955	7542	7203	3995	1499	1419	1051	614
6951	6063	4909	2513	11176	10595	6982	4882
1309	892	918	466	2105	1993	1298	681
**actin**							
C	100	200	500	C	100	200	500
8847	8934	9273	9403	10425	10695	10832	11021
5892	5938	5263	5509	9473	9566	8942	9892
8467	8122	7959	7821	10812	10253	9945	9898
